# Optical Tracking Data Validation and Orbit Estimation for Sparse Observations of Satellites by the OWL-Net

**DOI:** 10.3390/s18061868

**Published:** 2018-06-07

**Authors:** Jin Choi, Jung Hyun Jo, Hong-Suh Yim, Eun-Jung Choi, Sungki Cho, Jang-Hyun Park

**Affiliations:** 1 Center for Space Situational Awareness, Korea Astronomy and Space Science Institute, 776, Daedeokdae-ro, Yuseong-gu, Daejeon 34055, Korea; rutcome@kasi.re.kr (J.C.); yimhs@kasi.re.kr (H.-S.Y.); eunjung@kasi.re.kr (E.-J.C.); skcho@kasi.re.kr (S.C.); jhpark@kasi.re.kr (J.-H.P.); 2 Astronomy and Space Science Department, University of Science and Technology, 217, Gajeong-ro, Yuseong-gu, Daejeon 34113, Korea

**Keywords:** CCD, optical telescope, OWL-Net, short-arc, sparse data, orbit estimation

## Abstract

An Optical Wide-field patroL-Network (OWL-Net) has been developed for maintaining Korean low Earth orbit (LEO) satellites’ orbital ephemeris. The OWL-Net consists of five optical tracking stations. Brightness signals of reflected sunlight of the targets were detected by a charged coupled device (CCD). A chopper system was adopted for fast astrometric data sampling, maximum 50 Hz, within a short observation time. The astrometric accuracy of the optical observation data was validated with precise orbital ephemeris such as Consolidated Prediction File (CPF) data and precise orbit determination result with onboard Global Positioning System (GPS) data from the target satellite. In the optical observation simulation of the OWL-Net for 2017, an average observation span for a single arc of 11 LEO observation targets was about 5 min, while an average optical observation separation time was 5 h. We estimated the position and velocity with an atmospheric drag coefficient of LEO observation targets using a sequential-batch orbit estimation technique after multi-arc batch orbit estimation. Post-fit residuals for the multi-arc batch orbit estimation and sequential-batch orbit estimation were analyzed for the optical measurements and reference orbit (CPF and GPS data). The post-fit residuals with reference show few tens-of-meters errors for in-track direction for multi-arc batch and sequential-batch orbit estimation results.

## 1. Introduction

Space Situational Awareness (SSA) aims at detecting and predicting hazardous events by man-made and natural objects through ground-based or space-based sensors [1]. The United States operates the Space Surveillance Network (SSN), which consists of ground-based optical and radar sensors as well as space-based visible satellites [2]. Since 2008, the European Space Agency (ESA) has also implemented an SSA program for Space Surveillance and Tracking, Space Weather, and Near-Earth Objects. The already existing and available national sensors in Europe will be integrated together with newly developed elements [3]. The number of registered unclassified space objects exceeded 19,000 on 16 April 2018 [4].

The Korea Astronomy and Space Science Institute (KASI) has been developing an Optical Wide-field patroL-Network (OWL-Net) as part of the SSA facility of South Korea since 2010. The OWL-Net consists of five optical tracking sensors installed in Mongolia, Morocco, Israel, United States, and South Korea [5]. This global network of telescopes operates as a fully automatic sensor. Maintaining orbital ephemeris of 11 South Korean low Earth orbit (LEO) satellites is the top priority goal of the OWL-Net. A key feature of the OWL-Net is a chopper system designed to obtain dense astrometric measurements from single images from a charge-coupled device (CCD) sensor. The chopper rotates to cut a long trail into tens of small ones during the exposure time of the CCD sensor. We could obtain hundreds of astrometric measurements from a few images during a single optical observation chance at an OWL-Net site. The detailed specifications of the OWL-Net are described in the next section.

Optical observation data are short and sparse. This means that the observation time interval is much longer than the observation duration, while the observation duration lasts only a few minutes in a single-arc orbit of a satellite. Unlike other ground-based sensors, the optical sensor operates in a passive way to detect reflected sunlight during night time. Therefore, the possible observation times for optical tracking is limited to dawn and dusk for LEO satellites. These short-arc, sparse data can result in unstable solutions of orbit estimation [6]. Instead of a precise orbit estimation process, the short-arc, sparse data can be used to find initial orbit with uncorrelated targets [7]. Kim et al. [8] have described that unscented transformation and a particle filter can be suggested to solve the sparse data problem. An atmospheric drag coefficient estimation with a varying time span was an alternative solution to this problem [9,10]. However, the short-arc orbit determination strategy has been suggested as a more advantageous way for orbit determination and prediction in practical experiments [11]. The short-arc orbit determination study was conducted not only using the single laser tracking sensor but also using the optical tracking sensor [12]. Only two short-arc observation data were used for 2 or 3 days to support subsequent follow-up observations over a few days in Sang and Bennett’s study [12]. In the case of the OWL-Net, the orbit determination process was designed to run on a weekly basis.

Genova et al. [13] suggested another estimation strategy for the planetary mission spacecraft BepiColombo on the ESA mission to Mercury. The batch-sequential method was considered for the sequential updating of unmodeled dynamic parameters on behalf of the classic multi-arc method. Besides, improved dynamic parameters resulted in better estimates of the “global parameter”, such as the gravitational coefficient during next step multi-arc estimation. In the case of orbit estimation for LEO satellites with the OWL-Net, basically pre-defined dynamic models are used to estimate only state vectors (position and velocity) of target satellites. In some cases, atmospheric drag models do not fit well with actual in situ due to irregular solar activity. However, orbit determination with a short arc was unstable due to a priori uncertainty [14,15]. Therefore, we suggested a sequential-batch estimation strategy with short-arc, sparse optical observation data from the OWL-Net. The atmospheric drag coefficient was estimated sequentially a priori from the result of the multi-arc batch estimation. The sequential-batch estimation strategy and orbit determination results are described in Section 4.

In Section 2, we briefly describe the accuracy of the astrometric measurement from the optical observations of the OWL-Net. The astrometric measurements were compared with outer orbital ephemeris, Consolidated Prediction Format (CPF), and Precise Orbit Determination (POD) with onboard Global Positioning System (GPS) data. In the next section, the short-arc, sparse problem is analyzed for the OWL-Net using simulation data. Then, the sequential-batch estimation strategy is described with orbit determination using the actual astrometric data from the OWL-Net.

## 2. Astrometric Accuracy of the Optical Tracking Data for the OWL-Net

The OWL-Net is a fully automatic optical observation system. The OWL-Net consists of a headquarters and an on-site system. The Head-Quarter (HQ) system is responsible for sending daily schedules and processing orbital information maintenance. The Site Operation System (SOS) in each station operates individual optical tracking systems by a given observation schedule [16].

The optical tracking sensor consists of a 0.5-m telescope with 1.1-degree × 1.1-degree field of view. The pointing accuracy of the mount system is less than 10 arc seconds with a maximum speed of 2 degrees/s^2^. The back-end system of the OWL-Net consists of five parts. The imaging sensor is a PL16803 camera model by Finger Lakes Instrumentation LLC (FLI, Lima, NY, USA), which is a 4096 × 4096 back-illuminated CCD. The de-rotator compensates for the motion of the alt-az type mount. Blue-Visible-Red-Infrared-Clear (B-V-R-I-C) optical filters are used for photometric observation. The fourth part is a chopper wheel. The time-tagger system records the opening/closing time by the rotation of the chopper wheel [16,17]. A mechanical shutter also exists between the surface of the CCD and the chopper wheel.

**Figure 1:**
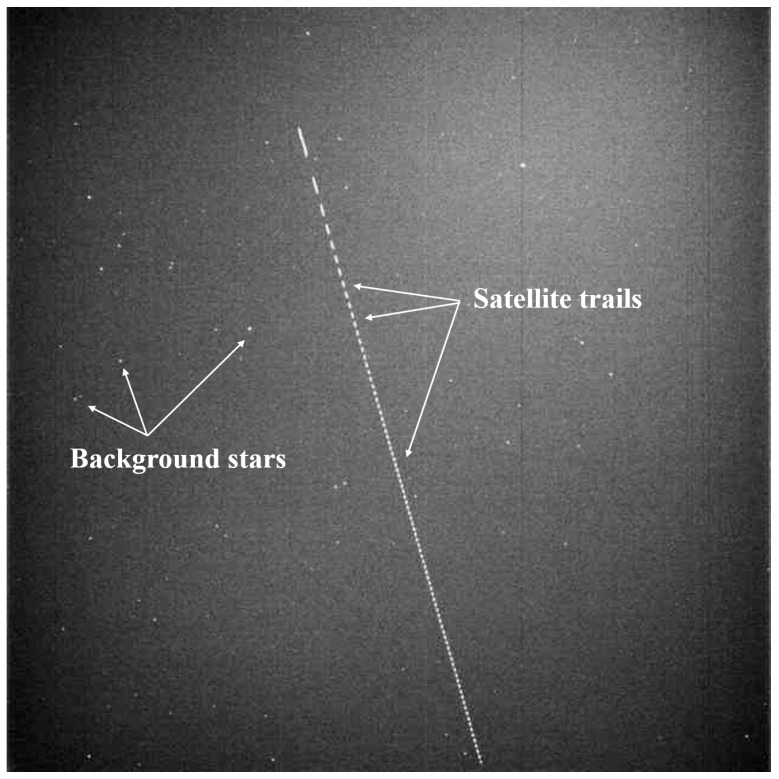
Optical observation image of CRYOSAT 2 on 1 June 2016 at the Israel site of the Optical Wide-field patroL-Network (OWL-Net). CRYOSAT 2 observed on sidereal tracking at the maximum chopper speed. The longer trails are the start of the observation.

In the LEO satellite optical observation mode of the OWL-Net, the mount system points to the same background stars during the shutter exposure time (i.e., sidereal tracking). The LEO satellite moves in a direction and speed against the stars on the celestial sphere. Reflected light from LEO satellites creates long trails on the surface of the CCD sensor. The chopper wheel makes several trails from a single image by chopping the original long trail. The maximum speed of the chopper wheel is 50 Hz, and the speed of the chopper wheel can be reduced to identify individual small trails more easily. Figure 1 shows an optical observation image of the CRYOSAT 2 satellite acquired at the Israel station of the OWL-Net on 1 June 2016. The chopper wheel accelerates to a constant rotation speed. Therefore, earlier recorded trails are longer than later ones.

The final measurements of the OWL-Net were topo-centric right ascension and declination [17]. The position of each trail was transformed to astrometric positions from physical coordinates on the surface of the CCD by using the corrected angular coordinates of stars in the same image. The World Coordinates System (WCS) solution tool was used with the General Star Catalogue (GSC) 1.2 [18].

**Figure 2:**
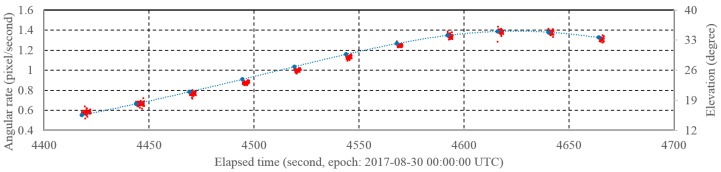
Angular rate (pixels/second) and pointing elevation (degree) for 11 shots in one observation set for CRYOSAT 2. The observation was performed at the OWL-Net in Israel. The angular rates in a single shot had similar values. The angular rates are proportional with the elevation angle of the satellite from the ground.

After the coordinate correction, the angular position of the trails and recorded time set were matched by the rate of change of each measurement. This was done in consideration of the physical acceleration motion of the chopper wheel. However, some errors occurred during this process because of false detection or software problems [19]. The errors could make problems for the matching of time and metric data. In that case, the erroneous matching affects the accuracy and precision of the orbit estimation. We also calibrated these errors using the rate of angular rate after normal data processing. Figure 2 shows the angular rate (pixels per second) of the optical observation for CRYOSAT 2 using the OWL-Net in Israel. The elevation angle of CRYOSAT 2 from the ground was also shown to understand the geometric condition of the optical observation. As the elevation angle was increased, the angular rates were also increased. However, the angular rates for each small trail in a single shot were maintained within small fluctuations. This means that the rate of the angular rates is almost consistent in the single shot. The whole optical observation dataset in this study was manually confirmed again.

The errors of the matching of time and metric data resulted irregular errors in the position of the satellite or the time slip in the optical measurements. In the case of orbit estimation with multi-arc observation, the errors result in bigger precision errors of the estimated orbit. However, the orbit estimator diverged from the short-arc optical observation without the calibration of the matching of time and metric data. Furthermore, time slip error growth occurred in the in-track direction error of the estimated orbit. The estimated parameters such as position, velocity, and atmospheric drag coefficient had bigger errors compared with the results of precise orbit estimation using SLR (Satellite Laser Ranging) or GPS data.

We conducted calibration and validation processes for the optical observation of LEO satellites during the development and test phase of the OWL-Net. The target LEO satellites were CRYOSAT 2 and KOMPSAT 5. CRYOSAT 2 was developed for monitoring changes in the thickness of the ice on the Earth. The average altitude of CRYOSAT 2 is about 720 km with 88 degrees of orbital inclination. CRYOSAT 2 was a good validation target with enough brightness and observation chances for the OWL-Net with a non-Sun-synchronous orbit. KOMPSAT 5 was selected as a validation observation target among Korean LEO satellites. It is operational on a Sun-Synchronous Orbit (SSO). KOMPSAT 5 was bright enough to detect using the telescope of the OWL-Net. Detailed orbital and physical characteristics of the two LEO satellites are described in Table 1.

**Figure 3:**
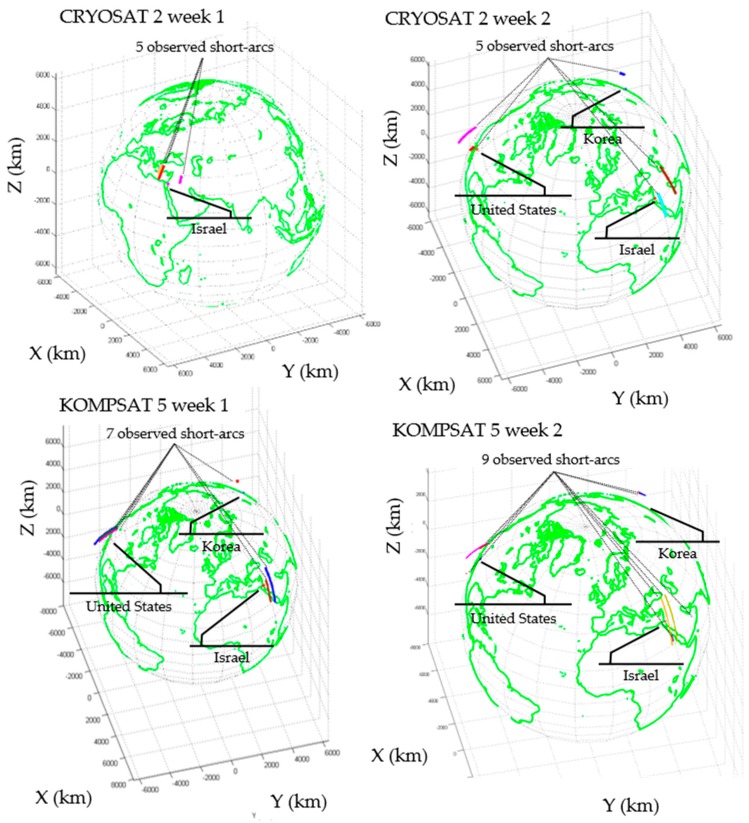
The three-dimensional (3D) orbit of CRYOSAT 2 and KOMPSAT 5 when the optical observations were performed by the OWL-Net. In Earth-Centered Fixed frame, the positions of the OWL-Net stations are marked with the names of the countries in which they were installed. The 3D orbit of CRYOSAT 2 and KOMPSAT 5 are presented as colored arcs.

We selected two sets of observation data for two separate weeks for CRYOSAT 2 and KOMPSAT 5. Table 2 shows the summary of the successful optical observation for CRYOSAT 2 and KOMPSAT 5. We obtained observations in Israel, the United States, and Korea. Due to weather conditions and system maintenance, the Mongolia and Morocco stations were not used. The observation duration indicates the time between the first and last observation point of each observation chance. The average observation duration was 2.4 min. The observation duration and number of shots counted were for successful observations only. The total numbers of observation points for CRYOSAT 2 were 1929 and 1041 for weeks 1 and 2, respectively. In the case of KOMPSAT 5, the total numbers of observation points were 1058 and 1255 for weeks 1 and 2. The falsely detected points were neglected and some observation points were not detected due to brightness or unknown reasons. Figure 3 shows the three-dimensional (3D) orbit of CRYOSAT 2 and KOMPSAT 5 when the optical observations were performed. In the case of the week 1 observation of CRYOSAT 2, the optical observation was performed at a single station in Israel.

The CRYOSAT 2 and KOMPSAT 5 optical observation data from the OWL-Net were compared with CPF and POD with onboard GPS data, respectively, taking the maneuvers into account. During the selected observation span, we confirmed that there were no maneuvers for CRYOSAT 2 and KOMPSAT 5. CRYOSAT 2 is one of the satellite laser ranging (SLR) observation target satellites. In the case of the SLR satellites, satellite predictions for laser tracking were provided in a CPF file via the International Laser Ranging Service (ILRS) webpage [20]. Generally, the accuracy of a CPF is a few tens of meters, which is more accurate than public Two Line Elements (TLEs) [21]. KOMPSAT 5 has a dual-frequency IGOR GPS receiver which is carried to obtain accurate positioning for synthetic aperture radar (SAR) missions [22]. The accuracy of the GPS receiver is within a few centimeters.

In the case of the CPF file, it included a 7-day prediction and was provided on a daily schedule. It indicated that a single CPF file could be used as a comparison file for 7 days of observation. However, we used the first day prediction only for each CPF file. It was concerned with the accuracy of prediction for the CPF file. The difference between the CPF file on other days had grown over time. We used 2-day prediction data from a single CPF file as a comparison when there was no CPF file for that day.

POD with onboard GPS data position information of the KOMPSAT 5 was provided using the SP3 format. This information was not prediction data but in situ measurements. However, some data were lacking for both of the observation weeks for KOMPSAT 5 with the OWL-Net. The second observation time for week 1 for KOMPSAT 5 lacked the POD with onboard GPS data. Therefore, we could not make the comparison for the second observation data of week 1 for KOMPSAT 5.

The ephemeris data was converted into identical frames as the optical observation data in consideration of the optical observation models. The CPF and onboard POD with GPS data were provided as a rectangular coordinates forms in Earth-Centered Fixed. Therefore, we converted CPF and POD with onboard GPS data to topo-centric angular coordinates in an Earth-Centered Inertial coordinate frame at J2000.0 with the location information of each site for each observation set. The observation models were also considered. The light travel time from satellite to the optical telescope was corrected. The annual aberration effects were also corrected [23]. Equations (1) and (2) describe light travel time and annual aberration effect, respectively [24]. In Equation (2), α is the true right ascension, $\overset{´}{\alpha }$ is the observed right ascension, δ is the true declination, $\overset{´}{\delta }$ is the observed declination, $\varepsilon_{T}$ is the true obliquity of date, and ω is the geocentric longitude of the Sun in the ecliptic plane.

(1) $$\mathrm{light}\;\mathrm{travel}\;\mathrm{time}\;\left(t\right)=\frac{\mathrm{range}\;\mathrm{from}\;\mathrm{satellite}\;\mathrm{to}\;\mathrm{optical}\;\mathrm{telescope}\;\left(R\right)}{\mathrm{speed}\;\mathrm{of}\;\mathrm{light}\left(c\right)}$$

(2) $$\alpha =\;\overset{´}{\alpha }-\frac{20.5(\cos\overset{´}{\alpha }\cos\omega \cos\varepsilon_{T}+\sin\overset{´}{\alpha }\sin\omega )}{\cos\overset{´}{\delta }}\;\delta =\;\overset{´}{\delta }-20.5\left(\cos\omega \cos\omega_{T}\left(\tan\varepsilon_{T}\cos\overset{´}{\delta }-\sin\overset{´}{\alpha }\sin\overset{´}{\delta }\right)+\cos\overset{´}{\alpha }\sin\overset{´}{\delta }\sin\omega \right)\;$$

**Figure 4:**
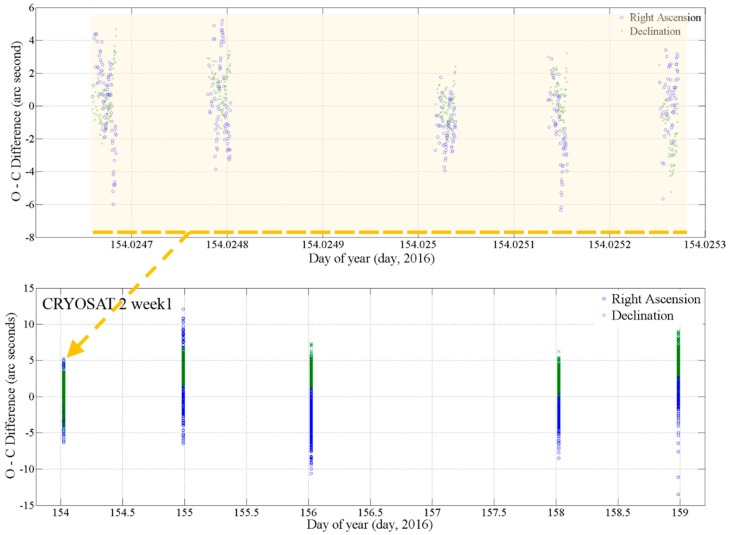
Residual observation-calculated (O-C) difference between the optical observation data from the OWL-Net and comparison Consolidated Prediction File (CPF) data for CRYOSAT 2 on 1 June 2016. The observation was performed by the OWL-Net at the Israel site. The upper graph shows the residual for 1 June 2016. There was one optical observation chance with five shots. The lower graph shows the residual from 1 June 2016 to 6 June 2016 for CRYOSAT 2.

The residual observation-calculated (O-C) difference is shown in the time sequence in Figure 4. For CRYOSAT 2, the upper graph in Figure 4 is the residual between the observation and the comparison from the observation on 1 June 2016. The optical observation was performed for five shots in one observation chance. We obtained tens of optical observation points from each shot. There were irregular patterns in each single shot. The pattern was caused by temporal shaking of the mount by wind or photometric error. The upper graph is for first date results in the lower graph. The lower graph shows the residual for the first week for CRYOSAT 2 in Table 2. In this week, we obtained five optical observation chances at the Israel site. The widths of difference were changed from 10 to 25 arc seconds, caused by the weather conditions and time-synchronization accuracy.

**Figure 5:**
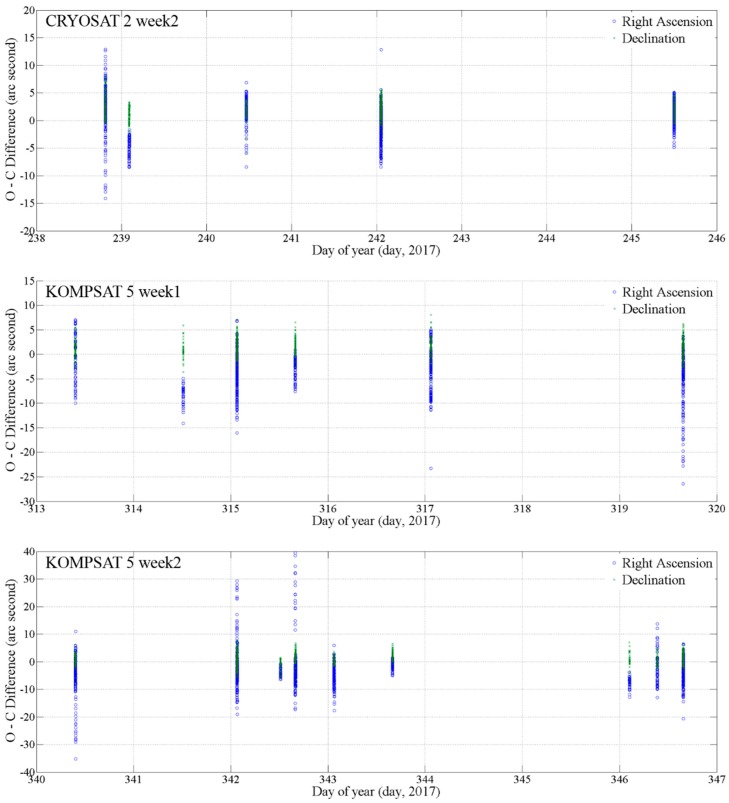
Residual O-C difference between the optical observation data from the OWL-Net and the comparison CPF data for CRYOSAT 2 in August 2017 for the first graph. The second and third graphs show the difference between the optical observation data from the OWL-Net and onboard Global Positioning System (GPS) data. The optical observation data for KOMPSAT 5 was performed in November 2017 and December 2017, respectively. The maximum difference for KOMPSAT 5 was bigger than the result for CRYOSAT 2.

Figure 5 shows the O-C difference for the rest of the three optical observation sets for CRYOSAT 2 and KOMPSAT 5. The first graph is for the result of the observation in week 2 for CRYOSAT 2. The width of the differences is similar to the result for week 1 in Figure 4. However, the widths of the differences for KOMPSAT 5 in the second and third graph are bigger than the differences for CRYOSAT 2. The optical observation errors were caused by the seeing condition for the weather and time-synchronization accuracy. In particular, the optical tracking system measured two-dimensional metric data on the celestial field. Therefore, the optical observation errors were increased with the satellite on a lower altitude.

**Figure 6:**
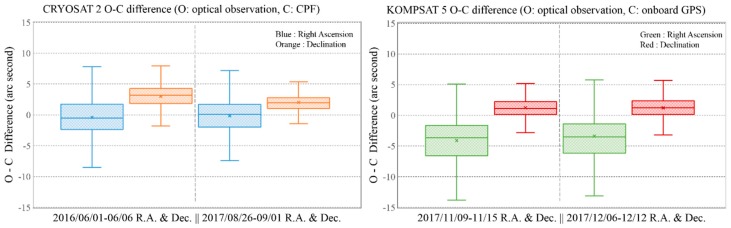
Box and whisker plot comparison for the 1-week O-C difference between optical observation data and precise orbit ephemeris (CPF and Precise Orbit Determination (POD) with onboard GPS data) for CRYOSAT 2 and KOMPSAT 5. The left graph for CRYOSAT 2 was the result of the comparison with CPF for 2 weeks in 2016 and 2017. The optical observation for KOMPSAT 5 in the right-side graph were compared with POD with onboard GPS data.

Statistical analysis of the O-C difference comparison was performed for two LEO satellites, CRYOSAT 2 and KOMPSAT 5, with the optical observation data for 2 weeks. In Figure 6, the left box and whisker plot comparison is an O-C difference analysis for CRYOSAT 2 and the right image shows the same for KOMPSAT 5. For each box and whisker plot, the “x” locates the mean value. The mean differences for right ascension for CRYOSAT 2 were −0.41 and −0.16 arc seconds, while the mean differences for declination for CRYOSAT 2 were 3.0 and 2.0 arc seconds. The absolute values for the differences of declination were larger than those for right ascension. In the case of KOMPSAT 5, the mean differences of right ascension were −4.1 and −3.9 arc seconds and the mean differences of declination were 1.2 and 1.2 arc seconds. The box shape for the bottom and top was made with the first and third quartiles. The height of each box is smaller than 5 arc seconds. However, the widths between whiskers is almost 20 arc seconds. The boxes and whiskers are symmetrical with a similar median and mean value. Even the maximum O-C difference for right ascension in Figure 5 is almost 40 arc seconds. However, these results are neglected as outliers in Figure 6. This indicates that the outliers are of negligible value for statistical analysis of the accuracy of the optical observation results of the OWL-Net.

There are two comparisons of the historical optical tracking system for SSA with the OWL-Net. The Baker-Nunn camera is the first-generation optical tracking network for LEO satellites and their launch vehicles. The along-track metric accuracy of a single Baker-Nunn camera is about 2 arc seconds in stationary mode with Very Large Frequency (VLF) signals time keeping [25]. The Baker-Nunn camera was replaced by Ground-Based Electro-Optical Deep Space Surveillance (GEODSS) [26]. The GEODSS is a dedicated optical sensor for observing deep space objects as part of the SSN. The sidereal metric observation accuracy of the GEODSS is 4–6 arc seconds. The accuracy of its time synch is within 0.001 s [27]. There is another optical tracking sensor contributing to the SSN. The Maui Space Surveillance System (MSSS) consists of multiple assets capable of providing highly accurate optical observations for missiles or satellites. In the case of the 1.6-m Advanced Electro-Optical System (AEOS), the accuracy of metric data varied from 2 arc seconds to more than 10 arc seconds for the LAGEOS 2 satellite. The altitude of LAGEOS 2 is about 1300 km. The Raven telescope has 0.4-m optics to operate with higher accuracy ballistics or rate-track methods. The Root-Mean-Square (RMS) pointing error magnitude is 2.2 arc seconds for the GPS optical observation compared to the true GPS ephemeris [28].

## 3. Characteristics of the Optical Observation Data with the OWL-Net: Dense Data in Short Arc between Sparse Observation Chances

The optical observations of satellites can be implemented by satisfying three constraints: site elevation angle, Sun elevation angle, and lighting condition. In the case of the OWL-Net, the site elevation angle is set to 15 degrees. This means that system tracks the target satellite when the target satellite is 15 degrees above the ground. The Sun elevation angle is set at −12 degrees at the astronomical twilight level. The penumbra and direct sunlight is considered as the lighting condition. Observation time span of the LEO satellite with the OWL-Net is limited to dawn and dusk. Even if prior constraints are satisfied, the read-out time of the CCD sensor and telescope pointing time are also considered in the actual observation schedule.

**Figure 7:**
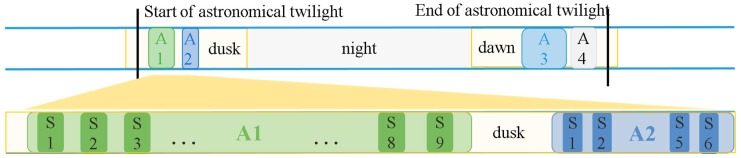
Optical observation schedule of the OWL-Net. Constraints of the observable time: Sun elevation angle (astronomical twilight, −12 degrees), target satellite elevation angle, and lighting condition (penumbra and direct Sun). During the observable time span (A1, A2, etc.), several shots (S1, S2, etc.) could be achieved with charge-coupled device (CCD) readout and mount moving time.

Figure 7 shows the example of the optical observation schedule during a single night and the optical observation constraints. We defined each observation chance as “Action” (A1, A2, etc.) and each shot as “Shot” (S1, S2, etc.), respectively. Generally, each LEO optical observation target had one or two actions per night for each OWL-Net site. The length of each action and the number of shots are dependent on the optical observation conditions as we previously described.

The optical tracking simulation with the OWL-Net was performed to analyze the length of the arc and interval between the optical observation chance under the optical tracking condition for the OWL-Net. The simulation targets were 11 South Korean LEO satellites. The average altitude from the ground was 730 km. Except for one target, the other targets have circular orbits. Among the 11 targets, eight targets were designed to have a Sun-synchronous orbit (SSO). The target satellites in SSO revisit the same location at the same local hour every day [29]. The optical observation chance can be reduced for the LEO satellites in SSO. The simulation was performed for one year during 2017. However, we did not consider the weather conditions and abnormal conditions of the system or maintenance of the system. As an example, the Mongolia site had been down for a few months due to the low temperature for operating hardware systems. In the development and testing phase of the OWL-Net, we faced many abnormal situations which forced us to improve the hardware or software of the system. In addition, we achieved observations of a bright launch vehicle for a system test in the early phases of the development of the OWL-Net.

**Figure 8:**
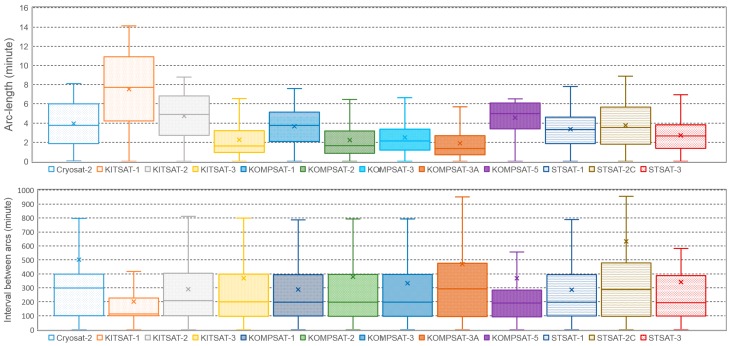
Statistics of the length of the arc and interval between observation chances for 11 LEO targets of the OWL-Net and CRYOSAT 2 from the optical observation simulation for 2017. The mean value of lengths of the arc (arc-length) did not exceed 4 min, whereas the mean value of intervals between the optical observation chance was about 400 min.

Figure 8 shows the results of the optical observation simulation of the OWL-Net. The mean length of the arc (arc-length) for a single optical observation chance is about 4 min. Except for KITSAT 1, the maximum time of the optical observation does not exceed 10 min. The average period of orbit for 12 LEO satellites is 100 min; therefore, the mean arc-length for optical observation is only 4–5% of the entire orbit. On the other hand, the mean value of the interval between the optical observation chances is about 400 min. The maximum interval of nine LEO satellites exceeds 800 min. This indicates that the optical observation using the OWL-Net for 12 LEO satellites can be performed for 4 min per four revolutions. However, in reality, some sites can be shut down due to weather conditions or maintenance. Practically, as shown in selected cases in Table 2, we were only able to perform the optical observation five times per week for CRYOSAT 2. The lower whiskers of the lower graph in Figure 8 mean that the satellite was observed simultaneously with relatively close stations.

The optical observation from the OWL-Net was performed in a very short duration with sparse intervals of the observation chance, but the observation data in the single shot was also dense. These high-rate optical observation data can be achieved with the chopper system as we previously described. Vallado et al. [30] considered that several hundred observation points per arc comprised “dense” data with the SSN sensor. The regular observation points of the SSN were achieved, averaging three points per arc. The OWL-Net’s high-rate, dense optical observations can help to make orbital estimations more stable with the short-arc optical observations.

## 4. Short-Arc Orbit Estimation with the Optical Observation Data of the OWL-Net

The batch least-squares (BLS) orbit estimation is a typical orbit determination method. Another orbit estimation method, the sequential filtering orbit estimation, is more flexible and able to provide a fast response for sequentially added observations. These two methods have advantages and disadvantages for measurement processing, computing power, divergence, and the influence of bad data, etc. The BLS orbit estimation needs more computing power and has a slower response speed than the sequential filtering. However, when the observation data are sparse and noisy, the BLS orbit estimation can be a more suitable answer for stable orbit estimation results [31].

The BLS orbit estimation solution is described as Equation (3), with x and *P* being determined a priori [32]. Here, x and *P* denote a state deviation vector and covariance, respectively. The BLS orbit estimator in this study was developed with a numerical orbit propagator using the special perturbation (SP) method [33].

(3) $${\tilde{x}}_{k}={\left(H^{T}WH+{\bar{W}}_{k}\right)}^{-1}\left(H^{T}Wy+{\bar{W}}_{k}{\bar{x}}_{k}\right),\;P={\left(H^{T}WH+{\bar{W}}_{k}\right)}^{-1}$$

In the case of the optical tracking observation, the angle-only measurements, right ascension and declination, are acquired without the range information. The OWL-Net is a ground-based optical sensor; therefore, a topo-centric environment condition was considered for the observation model. The measurements for satellites from the OWL-Net were corrected with the coordinates of stars in J2000.0 [18]. Therefore, the same coordinate system was used in the orbit estimation program. In Equation (4), the observation model for the optical tracking observation is described. The range from ground-based optical tracking sensor to target satellite, r, was calculated with the initial orbital information.

(4) $$\alpha =\;\tan^{-1}\frac{r_{2}}{r_{1}},\;\delta =\;\sin^{-1}\frac{r_{3}}{r}$$

The modeling of the perturbation by the atmospheric drag has errors due to the irregular variation of the atmosphere. In Equation (5), the atmospheric density, ρ, is calculated using the atmosphere models. $C_{d}$ denotes the atmospheric drag coefficient, which can be changed by the physical characteristics of space objects. In the case of plate, the atmospheric drag coefficient is 2.2. However, the satellites can have complicated shapes, with the exception of some geodesy satellites. To overcome the error of the atmospheric drag density models and the variation of the effective area by forcing the atmospheric perturbation, the atmospheric drag coefficient or ballistic coefficient, $\frac{C_{d}A}{m}$, can be estimated. In this study, we estimated the atmospheric drag coefficient with the position and vector of the satellite to overcome the sparseness of the optical observation dataset for 7 days.

(5) $${\vec{a}}_{drag}=\;-\frac{1}{2}\frac{C_{d}A}{m}\rho v_{rel}^{2}\frac{{\vec{v}}_{rel}}{\left|{\vec{v}}_{rel}\right|}$$

The estimation of the atmospheric drag coefficient could be found with various intervals. The frequency of the estimation of the atmospheric drag coefficient affected the accuracy of the orbit estimation results [8,9,34,35]. The atmospheric drag coefficient was estimated at intervals of 1, 2, 8, 12, and 24 h in the research. In general, more frequent drag coefficient estimations improve orbit estimation precision. In the case of orbit estimation with the OWL-Net, the optical observation data were too sparse and a single action was too short to estimate the atmospheric drag coefficient within a fixed time interval. This is because optical observation events cannot occur repeatedly at regular intervals and cycles. On the other hand, too-short arc optical observations can result in filter divergence or inaccurate orbit estimation results [36]. Accordingly, we sequentially estimated the atmospheric drag coefficient for each single action with short-arc BLS orbit estimations a priori from the estimated orbital parameter by multi-arc BLS orbit estimation results.

**Figure 9:**
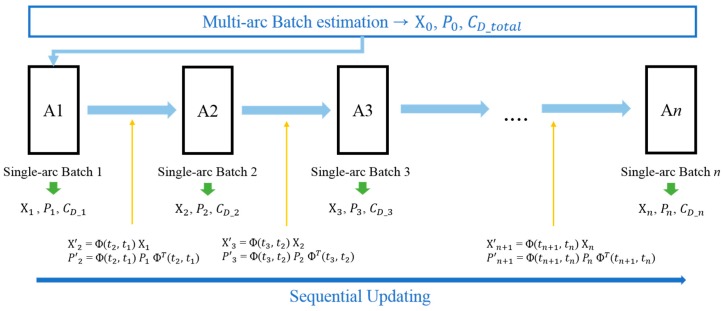
Sequential-batch orbit estimation diagram. In the multi-arc batch orbit estimation step, the position and velocity vectors and the single atmospheric drag coefficient are estimated by optical observation data epochs. After that, batch orbit estimations for single short-arcs are conducted by sequentially estimating the atmospheric drag coefficient. The position, velocity, and covariance were propagated to the next short arcs with results from the present short-arc batch orbit estimation.

In Figure 9, the sequential-batch least-squares orbit estimation is described. First, multi-arc batch least-squares orbit estimation is performed using the optical observation data for 7 days in accordance with the orbit determination strategy of the OWL-Net system. The position and velocity information from TLE was employed before this step. After that, the short-arc batch least-squares orbit estimations for each single short-arc action is conducted sequentially with the re-estimation of the position and velocity and the atmospheric drag coefficient. The estimation result from the multi-arc batch orbit estimation is used a priori for the first short-arc batch least-squares orbit estimation. By the second step of re-estimating the atmospheric drag coefficient of each single short-arc, the variation of the atmospheric drag coefficient can be achieved.

For the first multi-arc BLS orbit estimation, the position and velocity are calculated a priori with TLE in Equation (6). We selected TLE in consideration of backward propagation. TLE information was well fitted to the actual orbit within 1 or 2 days before the epoch of TLE [37]. The atmospheric drag coefficient for the first step was 2.2, which is a well-known arbitrary constant.

(6) $$X_{initial}=\left[x,y,z,v_{x},v_{y},v_{z}\right]\;from\;\mathrm{TLE}\;\left(C_{D}=2.2\right)$$

The estimated orbit parameter from the multi-arc BLS orbit estimation, $X_{0},\;P_{0}$, was determined before the single short-arc BLS orbit estimation for the first optical observation, Action 1. The estimated orbit parameters, $X_{1},\;P_{1}$, for the first short-arc action, Action 1, were propagated to the first optical observation point of the next short-arc action, Action 2.

(7) $${{Χ}^{\prime }}_{n+1}=\Phi \left(t_{n+1},\;t_{n}\right)\;{Χ}_{n}\;{P^{\prime }}_{n+1}=\Phi \left(t_{n+1},\;t_{n}\right)\;P_{n}\;\Phi^{T}(t_{n+1},t_{n})$$

The propagated state vector and covariance, ${X^{\prime }}_{1},\;{P^{\prime }}_{1}$, were used for the next short-arc BLS orbit estimation a priori in Equation (7). Finally, we obtained the estimated position and velocity, $\left[X_{1},\;P_{1}\right]$, $\left[X_{2},\;P_{2}\right]$, … $\left[X_{n},\;P_{n}\right]$, and the estimated atmospheric drag coefficients, $C_{D\_1}$, $C_{D\_2}$, … $C_{D\_n}$, for each single short-arc optical observation data point.

The BLS orbit estimator was developed for sequential-batch type orbit estimation. MATLAB software was used to build it. Table 3 shows the dynamic models and estimator specification. To overcome the truncation error in the calculation of high-order term geopotential perturbation (up to 60 × 60), the recursive computation of Legendre polynomials technique was applied [38]. We selected the Jacchia-Bowman 2008 (JB2008) atmosphere model to calculate the atmosphere density. The JB2008 model is an effective model to calculate the atmosphere density for irregular solar activity [39]. The shape of the estimated satellite was assumed to be a simple sphere model. The estimator was developed in a weighted BLS type. TLEs were collected a priori using a space-track website.

We considered four perturbations to describe the orbits of CRYOSAT 2 and KOMPSAT 5. The perturbations for space objects in Earth orbit have different scales by an altitude of the space objects [31]. The main perturbations were considered for this study. Relatively small scale perturbations like relativity and albedo were neglected. In particular, we focused on the atmospheric drag perturbation in this study because it varied due to not only the altitude of space objects but also the status of the atmosphere. The error by modeling of the atmosphere presented as the variation of the atmospheric drag coefficient.

**Figure 10:**
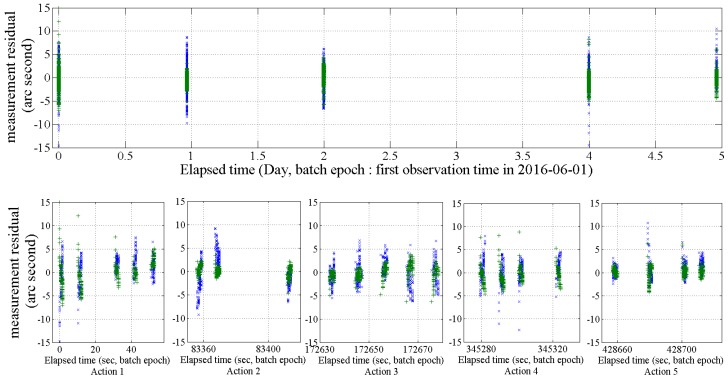
Measurement residuals of the multi-arc batch least-squares orbit estimation results (upper) and five single short-arc batch least-squares orbit estimation results (lower) with the optical observation using the OWL-Net at the Israel site for CRYOSAT 2. The optical observation was peformed from 1 June 2016 to 6 June 2016. Each short-arc optical observation was performed in 40–70 s. (blue cross: right ascension, green cross: declination).

The measurement residuals of multi-arc batch least-squares orbit estimation and sequential-batch least-squares orbit estimation are shown in Figure 10. The target satellite was CRYOSAT 2. The optical observation was performed using the OWL-Net at the Israel site from 1 June to 6 June 2016. The upper graph shows the measurement residuals for multi-arc BLS orbit estimation results. Both right ascension (blue cross) and declination (green cross) are within 15 arc seconds. The lower five graphs show the measurement residuals for the result of short-arc orbit estimation with five short-arc optical observations. Each of the optical observation durations was within 40–70 s. Single short-arc BLS orbit estimation was also successfully performed. The measurement residuals for short-arc BLS orbit estimation were similar to the multi-arc BLS orbit estimation results.

**Figure 11:**
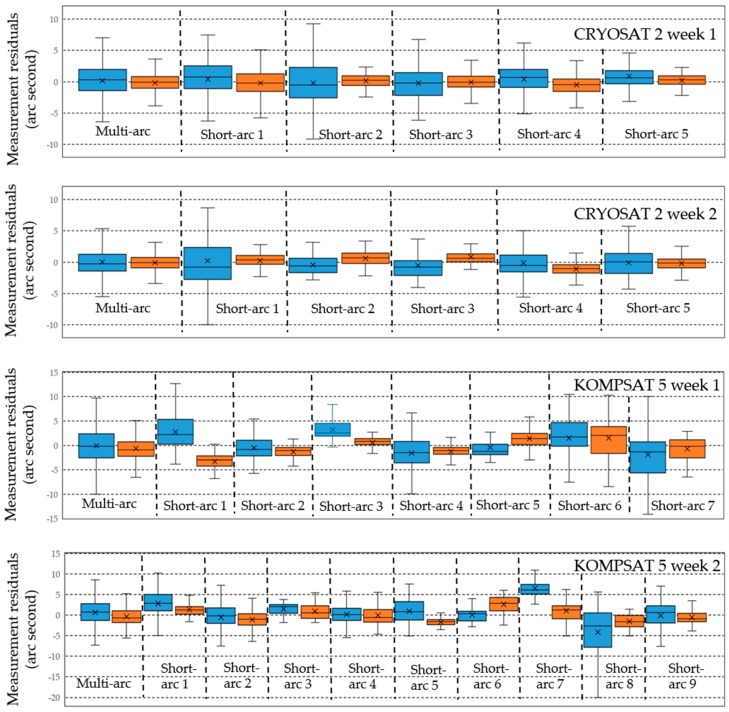
Measurement residuals of multi-arc batch least-squares orbit estimation results and short-arc batch least-squares orbit estimation results for CRYOSAT 2 and KOMPSAT 5 (blue: right ascension, orange: declination). The outliers were neglected. The mean values of the residuals of the multi-arc observations show smaller variation than the mean values of the short-arc observations. The mean values of most residuals do not exceed 5 arc seconds.

The measurement residuals of multi-arc batch least-squares orbit estimation and sequential-batch least-squares orbit estimation for four sets of CRYOSAT 2 and KOMPSAT 5 observations are shown as box and whisker plots in Figure 11 (blue: right ascension, orange: declination). The outliers were neglected in those graphs. In general, the residuals from the results using multi-arc orbit estimation were more stable than the residuals from the results using single short-arc orbit estimation. The residuals for KOMPSAT 5 were bigger and more unstable than the results of CRYOSAT 2. The mean values of the residuals of the multi-arc observations show smaller variation than the mean values of the short-arc observations. However, the mean values of most residuals do not exceed 5 arc seconds.

Post-fit residuals for CRYOSAT 2 and KOMPSAT 5 orbit estimation results are shown in Table 4. This table also lists the root-mean-squares error of the results of orbit estimation. The detailed observation information is summarized in Table 2. The post-fit residuals were calculated for both right ascension and declination. There were some result deficiencies due to the number of actions. The post-fit residuals for multi-arc BLS orbit estimation results were similar to the results from short-arc BLS orbit estimation. Some of post-fit residuals were bigger than others for the same week and satellite. In that case, the optical observation data presented larger irregular errors as we described in Section 2. The residuals for KOMPSAT 5 are relatively bigger than those of CRYOSAT 2 for the same reason.

The estimated atmospheric drag coefficients are shown in Table 4. In general, the atmospheric drag coefficient was fixed at 2.2 as we described earlier. However, the estimated atmospheric drag coefficient value can be reduced or increased by inaccurate values of a given mass or effective area of a satellite for orbit estimation. On the other hand, the estimated atmospheric drag also can be varied due to the error of the atmosphere model. The atmospheric drag coefficient for multi-arc observation was estimated with the optical observation data from the first point of the first short-arc observation to the last point of the last short-arc data. This indicated that the estimated atmospheric drag coefficient was an average value for 1 week. In another example, we used single short-arc data points to estimate the atmospheric drag coefficient for each short-arc. The estimated atmospheric drag coefficients for the short arc were average values only for single short arcs.

CRYOSAT 2 and KOMPSAT 5 were selected as the calibration and validation targets for the OWL-Net at the first observation test phase due to their provided precise ephemeris. As previously described in Section 2, CPF and onboard GPS data were provided. The provided ephemeris was used as a reference for comparing the estimated orbits. The post-fit residuals with reference orbit for CRYOSAT 2 and KOMPSAT 5 were analyzed for the estimated results of the multi-arc and short-arc BLS orbit estimations, respectively. In the case of the short-arc BLS orbit estimation, the estimated orbits were propagated from the epoch of each short arc to the epoch of the next short arc. Table 5 shows the post-fit residuals with reference results in the radial, in-track, and cross-track (RIC) frame. Comparison results for multi-arc and short-arc approaches were similar to each other for the RIC frame.

The post-fit residuals with reference show the accuracy of the estimated orbits of CRYOSAT 2 and KOMPSAT 5 with the optical observation data from the OWL-Net. The in-track direction error was bigger than the radial and cross-track direction errors. The errors of week 1 of CRYOSAT 2 were twice as large as the errors of week 2. In particular, the cross-track error was almost 10 times greater than the results of week 1. Both multi-arc and short-arc approaches were shown to have the same results. In the case of week 1, we employed the observation data using the OWL-Net in Israel only. In addition, the observations for each short arc were performed within 30 min on either side of midnight in Coordinated Universal Time (UTC). On the other hand, for week 2 of CRYOSAT 2, we obtained observations with the OWL-Net in Korea, Israel, and the United States. It was shown that using single-site tracking data causes incorrect orbit estimation results, especially for the cross-track direction.

In the case of the post-fit residuals with reference for KOMPSAT 5, the width of errors for the RIC frame was smaller than the post-fit residuals for CRYOSAT 2. We used CPF and POD with onboard GPS data for comparison with CRYOSAT 2 and KOMPSAT 5, respectively. In consideration of the offering accuracy of both references, the results of the KOMPSAT 5 were more reliable. When the space object was 600–2000 km above the ground, 1 arc second could be converted into 3–10 m. For the O-C difference in Section 2, the average O-C differences of KOMPSAT 5 were 3.5 and 1.2 arc seconds for right ascension and declination, respectively.

The post-fit residuals with reference to the multi-arc BLS orbit estimation results were similar to the short-arc BLS orbit estimation results. The multi-arc BLS orbit estimations were performed with whole short-arc sets. However, the short-arc BLS orbit estimations only used single short-arc data points to estimate the orbit and the atmospheric drag coefficient. Therefore, the errors could be increased because the estimated orbits with the short arc for a few minutes were propagated to the next optical observation dataset.

**Figure 12:**
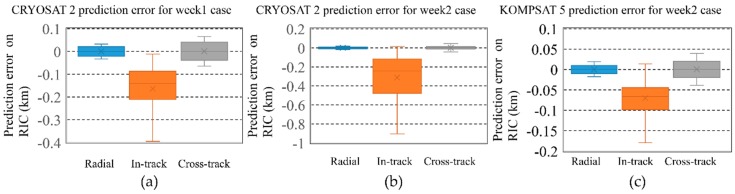
Prediction error for 7 days after multi-arc batch least-squares orbit estimation using 2 weeks of data for CRYOSAT 2 (**a**,**b**) and 1 week of data for KOMPSAT 5 (**c**). In one case for KOMPSAT 5, there was a maneuver event one day after the optical observation. The maximum prediction errors of two satellites did not exceed 1 km for 7 days.

The prediction errors with reference were analyzed for CRYOSAT 2 and KOMPSAT 5 in Figure 12. In the case of KOMPSAT 5, there was a maneuver event one day after the optical observations of the week 1 case. After one day, the in-track direction errors between POD with onboard GPS data and the prediction were dramatically increased to 10 km. We confirmed the maneuver event by comparing several consecutive TLEs with each other. The multi-arc BLS orbit estimation results were used to make predictions for 7 days. This was followed by a basic policy for the operation of the OWL-Net. The propagated orbits with the consecutive TLEs were also compared with CPF and POD with onboard GPS data. The in-track direction prediction errors showed a maximum 2.2 km of vibration. Occasionally, the maximum in-track direction error was increased to 5–6 km for KOMPSAT 5. The in-track direction errors were dominant in the 7-day prediction error for CRYOSAT 2 and KOMPSAT 5. The maximum in-track direction errors from the multi-arc BLS orbit estimations did not exceed 1 km for both satellites.

The range scale errors were not directly proportional to the astrometric scale errors. The range between the observer and KOMPSAT 5 varied from 550 to 2000 km within a single optical observation chance. The in-track direction error of 1 km can be converted to the astrometric scale error from 1.75 to 6.25 arc minutes in accordance with the range variation. The field-of-view (FOV) of the OWL-Net is 66 arc minutes. Another point of view for the in-track error is time synchronization. The speed of KOMPSAT 5 is 7.6 km per second. KOMPSAT 5 orbits 1 km in about 130 ms. The recorded time scale of the OWL-Net is one millisecond. Therefore, KOMPSAT 5 and CRYOSAT 2 can be tracked with enough accuracy of time and telescope pointing prediction for the subsequent 7 days with the orbit estimation results using the OWL-Net.

## 5. Discussion and Conclusions

The OWL-Net, the optical tracking network for SSA, is undergoing a testing phase in 2018. We performed calibration and validation for the optical observation performance using the OWL-Net for two selected LEO satellites, CRYOSAT 2 and KOMPSAT 5. The mean astrometric accuracy for right ascension and declination was within 5 arc seconds. The optical observation data for CRYOSAT 2 was compared with the CPF file. The reference orbit to which the optical observation data of KOMPSAT 5 was compared was POD with onboard GPS data in a normal situation. Occasionally, the astrometric error jumped to 40 arc seconds. The time synchronization was suspected to be a main error source. Because the optical observation data from the OWL-Net is very dense due to the chopper, maximum 50 Hz, some errors were neglected during the orbit estimation process.

The overall mismatching of time and position information caused large errors of accuracy for orbit estimation and filter divergence. The critical reasons for the mismatching of time and position information were false and mis-detections of trails. The detection parameters of the CCD sensor can affect the detection rate of the trails and the precision of the astrometry. In this study, we mainly considered the calibration of the matching of time and position. The mismatching problem was successfully corrected using the assessment of the angular rate of the satellites. We did not use any orbital information to correct the mismatching error.

Even though the optical observation data from the OWL-Net is very dense, it is generated within a very short duration of a few minutes. In addition, the optical observation chances are sparse. We performed the optical observation simulation for the OWL-Net to analyze the short-arc and sparse condition problems for 2017 with consecutive TLEs. The average observation duration for a single optical observation time is 4 min, while the average optical observation time span is 400 min. This sparse problem is more serious with actual weather conditions. Sometimes we failed to make an optical observation for over 10 days for a single LEO satellite. Additional optical stations or collaboration with other sensors could be a solution for this issue. In particular, the stations in the Southern Hemisphere of the Earth are good for simultaneous observation in a single orbit of a LEO satellite with the OWL-Net stations in the Northern Hemisphere.

The orbit estimation filter was divergent or the estimation results were not reliable due to the short duration of the optical observation data. In addition, the sparseness of the optical observation data limited frequent estimation for the atmospheric drag coefficient. Therefore, we adopted the sequential-batch least-squares orbit estimation strategy for more frequent estimations of the atmospheric drag coefficient.

The orbit estimation test was performed with the optical observation data during 1 week for CRYOSAT 2 and KOMPSAT 5. The sequential-batch type orbit estimation strategy was tested to estimate the atmospheric drag coefficient frequently using dense and sparse optical observation data. The batch orbit estimation for short arcs was performed a priori using the results of the multi-arc batch orbit estimation. The estimated orbit with each short-arc optical observation data point was propagated to the start point of the next short arc. From this estimation strategy, the short-arc batch estimations were conducted without filter divergence. The short-arc batch orbit estimations results show similar post-fit residual values to those of the multi-arc batch orbit estimation, as shown in Table 3. The post-fit residuals with reference orbit from CPF and POD with onboard GPS data also have similar results to those of the multi-arc and short-arc batch orbit estimations in Table 4.

In this study, the atmospheric drag coefficient was re-estimated for each short-arc-based estimated value from the multi-arc orbit estimation. The atmospheric drag coefficient variations for CRYOSAT 2 and KOMPSAT 5 were confirmed. The variations of the atmospheric drag coefficient can be simply confirmed using the TLE data. The orbital information from TLE is a mean value from the orbit estimation using the last few days from the epoch of each TLE. Therefore, variation of the ballistic coefficients from the TLE has similar sensitivity. However, the variation of the atmospheric perturbation calculated with the estimated atmospheric drag coefficients for short arcs shows a similar tendency with ballistic coefficients from TLEs.

The orbit prediction performance of the estimated orbit with the OWL-Net was confirmed with the reference orbits. The in-track direction errors did not exceed 1 km for 7 days. The purpose of determining the orbit of LEO satellites using the OWL-Net is to maintain the orbital information of Korean LEO satellites. Therefore, orbit estimation should provide accurate orbit information for tracking LEO satellites. Generally, TLEs are used to track space objects but may present errors within a few kilometers. In this light, the orbit estimation results in this study can support more accurate optical observations than TLEs for the OWL-Net.

## Figures and Tables

**Table 1:** Orbital and physical characteristics of low Earth orbit (LEO) targets.

Name	NORAD ID	Orbital Information	Launch Mass	Size
CRYOSAT 2	36508	altitude: 720 km inclination: 88 degrees non-Sun-synchronous	720 kg	4.6 m × 2.3 m
KOMPSAT 5	39227	altitude: 550 km inclination: 97.6 degrees Sun-synchronous	1400 kg	3.7 m × 2.6 m

**Table 2:** Summary of optical observation results for validating astrometric angular accuracy. The week number was used to separate the observation data by week for orbit determination. The observation duration and number of shots were counted for the successfully achieved optical observations.

Satellite	Week Number	Site	Date (yyyy-mm-dd)	Observation Duration (minute)	Number of Shot	Number of Point
CRYOSAT 2	1	Israel	2016-06-01	0.9	5	355
		Israel	2016-06-02	1.0	3	346
		Israel	2016-06-03	1.1	5	429
		Israel	2016-06-05	1.1	4	385
		Israel	2016-06-06	1.0	4	409
	2	Korea	2017-08-26	1.6	5	146
		Israel	2017-08-27	3.2	3	81
		USA	2017-08-28	0.8	3	106
		Israel	2017-08-30	4.1	11	516
		USA	2017-09-02	4.5	4	192
KOMPSAT 5	1	Korea	2017-11-09	0.5	2	70
		USA	2017-11-10	6.1	5	201
		USA	2017-11-10	0.05	1	43
		USA	2017-11-11	4.5	13	323
		Israel	2017-11-11	3.2	5	96
		USA	2017-11-13	4.4	7	157
		Israel	2017-11-15	5.1	6	168
	2	Korea	2017-12-06	0.9	3	127
		USA	2017-12-08	4.6	9	237
		USA	2017-12-08	0.05	1	41
		Israel	2017-12-08	5.5	9	263
		USA	2017-12-09	1.0	4	163
		Israel	2017-12-09	0.8	3	77
		Israel	2017-12-12	0.04	1	36
		Korea	2017-12-12	0.8	2	58
		Israel	2017-12-12	5.6	10	253

**Table 3:** The batch least-squares orbit estimator setup and dynamic models to calculate perturbations. The Jacchia-Bowman 2008 model was used as the atmospheric drag model to reduce the error of models by irregular solar activity.

**Dynamic models**	Earth‘s gravity	EGM2008 (70 × 70)	Pavlis et al. [40] Kuga and Carrara [38]
	Planetary	Sun and Moon, planets	Standish et al. [41]
	Atmospheric drag	JB2008, spherical shape	Bowman et al. [39]
	SRP	spherical shape	
**Estimator**	Integrator	RKF7(8)	
	Filter	Weighted batch least-squares	Long et al. [33]
	A priori	TLE	www.space-track.org [42]

**Table 4:** Post-fit residuals (Root-Mean-Square (RMS), arc seconds) of multi-arc and short-arc batch least-squares (BLS) orbit estimation results for CRYOSAT 2 and KOMPSAT 5 (right ascension (R.A.), declination (Dec.)). The short-arc BLS orbit estimation results were similar to the multi-arc BLS orbit estimation results. The estimated atmospheric drag coefficients ($C_{D})$ for multi-arc and short-arc BLS orbit estimations for CRYOSAT 2 and KOMPSAT 5 are described.

Satellite	Week Number	RMS (``)	Multi-arc	Short-arc1	Short-arc2	Short-arc3	Short-arc4	Short-arc5	Short-arc6	Short-arc7	Short-arc8	Short-arc9
**CRYOSAT 2**	1	R.A.	2.86	3.18	3.48	2.57	2.51	2.20	-	-	-	-
		Dec.	1.80	2.75	1.09	1.45	1.76	1.45	-	-	-	-
		$C_{D}$	1.882	1.885	1.892	1.889	1.910	1.886	-	-	-	-
	2	R.A.	2.73	4.81	1.52	2.45	2.31	2.03	-	-	-	-
		Dec.	1.65	1.18	1.30	1.59	2.06	1.12	-	-	-	-
		$C_{D}$	1.984	1.979	1.972	1.979	1.956	1.973	-	-	-	-
**KOMPSAT 5**	1	R.A.	4.2	4.26	4.68	2.42	3.75	3.71	2.31	4.66	-	-
		Dec.	2.57	2.66	3.75	1.76	1.95	1.87	2.49	3.49	-	-
		$C_{D}$	2.656	2.641	2.641	2.640	2.632	2.633	2.648	2.660	-	-
	2	R.A.	4.03	4.39	4.17	2.09	3.55	3.04	1.64	6.85	7.25	3.75
		Dec.	2.29	1.98	2.36	2.32	2.39	1.89	3.37	2.81	2.28	1.80
		$C_{D}$	2.852	2.840	2.839	2.837	2.839	2.847	2.851	2.849	2.850	2.855

**Table 5:** Post-fit residuals (meter) with reference of multi-arc and short-arc batch least-squares (BLS) orbit estimation results for CRYOSAT 2 and KOMPSAT 5. The references are CPF and onboard GPS data for CRYOSAT 2 and KOMPSAT 5, respectively. The post-fit residuals for short-arc BLS orbit estimation were calculated with propagation from the epoch for each short arc to the epoch of the next short arc.

Satellite	Week Number	Multi-Arc			Short-Arc with Integration		
		Radial (m)	In-Track (m)	Cross-Track (m)	Radial (m)	In-Track (m)	Cross-Track (m)
**CRYOSAT 2**	1	21.03	85.94	57.02	21.19	86.09	55.84
	2	11.13	40.67	5.25	11.45	39.44	5.68
**KOMPSAT 5**	1	4.14	13.83	8.28	4.54	17.24	8.07
	2	5.05	21.52	4.15	5.30	21.77	3.78
